# Melatonin alleviates morphine analgesic tolerance in mice by decreasing NLRP3 inflammasome activation

**DOI:** 10.1016/j.redox.2020.101560

**Published:** 2020-04-29

**Authors:** Qianjin Liu, Ling-Yan Su, Chunli Sun, Lijin Jiao, Ying Miao, Min Xu, Rongcan Luo, Xin Zuo, Rongbin Zhou, Ping Zheng, Wei Xiong, Tian Xue, Yong-Gang Yao

**Affiliations:** a Key Laboratory of Animal Models and Human Disease Mechanisms of the Chinese Academy of Sciences & Yunnan Province, Kunming Institute of Zoology, Kunming, Yunnan, 650223, China; b Kunming College of Life Science, University of Chinese Academy of Sciences, Kunming, Yunnan, 650204, China; c State Key Laboratory of Genetic Resources and Evolution, Kunming Institute of Zoology, Chinese Academy of Sciences, Kunming, Yunnan, 650223, China; d KIZ/CUHK Joint Laboratory of Bioresources and Molecular Research in Common Diseases, Kunming Institute of Zoology, Chinese Academy of Sciences, Kunming, Yunnan, 650223, China; e Hefei National Laboratory for Physical Sciences at the Microscale, School of Life Sciences, Division of Life Sciences and Medicine, University of Science and Technology of China, Hefei, Anhui, 230026, China; f CAS Center for Excellence in Brain Science and Intelligence Technology, Chinese Academy of Sciences, Shanghai, 200031, China

**Keywords:** Analgesic tolerance, Melatonin, Morphine, NLRP3 inflammasome, Pain, ASC, apoptosis-associated speck-like protein containing CARD, BSA, bovine serum albumin, CTSB, cathepsin B, DAPI, 4′, 6-diamidino-2-phenylindole, DMEM, Dulbecco’s modified Eagle’s medium, ELISA, enzyme-linked immunosorbent assay, GAPDH, glyceraldehyde-3-phosphate dehydrogenase, IBA-1, ionized calcium binding adapter molecule 1, IL-1β, Interleukin 1β, LDH, lactate dehydrogenase, LPS, lipopolysaccharide, NLRP3, NOD-like receptor protein 3, NMDAR, N-methyl-d-aspartate receptors, PBS, phosphate-buffered saline, PFC, prefrontal cortex, PKC, protein kinase C, ROS, reactive oxygen species, siNC, negative control siRNA, si*Nlrp3*, *Nlrp3* siRNA, WT, wild type

## Abstract

Morphine is frequently used for pain relief, but long-term morphine therapy in patients with chronic pain results in analgesic tolerance and hyperalgesia. There are no effective therapeutic treatments that limit these detrimental side effects. We found pretreatment with melatonin could decrease morphine-induced analgesic tolerance. There was a significant activation of the NLRP3 inflammasome in the prefrontal cortex and the peripheral blood of morphine-treated mice compared to control animals, which could be blocked by melatonin. The inflammasome activation induced by morphine was mediated by the microglia. SiRNA knockdown or pharmacological inhibition of the NLRP3 abolished the morphine-induced inflammasome activation. Co-administration of melatonin and low-dose morphine had better analgesia effects in the murine models of pain and led to a lower NLRP3 inflammasome activity in brain tissues. Mice deficient for *Nlrp3* had a higher nociceptive threshold and were less sensitive to develop morphine-induced analgesic tolerance and acetic acid-induced pain relative to wild-type animals. Concordantly, we observed a significantly elevated level of serum IL-1β, which indicates an increase of NLRP3 inflammasome activity associated with the reduced level of serum melatonin, in heroin-addicted patients relative to healthy individuals. Our results provide a solid basis for conducting a clinical trial with the co-administration of melatonin and morphine for the relief of severe pain.

## Highlights

• Melatonin blocks murine morphine analgesic tolerance via NLRP3 inflammasome.
• Melatonin eliminates excessive ROS and CTSB induced by morphine.
• Deficiency of *Nlrp3* salvages morphine analgesic tolerance.
• Melatonin combined with a low dose of morphine can be used for relieving pain.

## 1 Introduction

Pain is a natural response to injury and a major health problem affecting the quality of life for many people. Opioid analgesics, such as morphine, are essential for treating severe, perioperative and chronic pain [1,2]. However, long-term use of morphine can result in analgesic tolerance, in which analgesic efficacy gradually decreases at fixed drug doses, along with the development of paradoxical hyperalgesia [3,4]. Tolerance and hyperalgesia are the two main detrimental side effects of morphine treatment and severely limits the clinical usage of the drug [5].

Accumulating evidence suggests that neuroinflammatory are critical for the morphine induced analgesic tolerance and hyperalgesia [6,7]. The NOD-like receptor protein 3 (NLRP3) inflammasome is the best-studied inflammasome which composed of the NLRP3 sensor, caspase-1 and the adaptor molecule apoptosis associated speck-like protein containing a caspase recruitment domain (ASC) [8,9]. Upon NLRP3 inflammasome activation, matured caspase-1 mediates Pro-IL-1β cleavage into mature IL-1β and secretion [8,10]. Dysregulation of the NLRP3 can lead to autoimmune diseases, neurodegenerative diseases, multiple sclerosis and metabolic disorders [11]. Previous study showed that morphine paradoxically prolongs neuropathic pain in rats by amplifying spinal NLRP3 inflammasome activation [12]. However, whether the NLRP3 inflammasome participates in morphine analgesic tolerance is still unclear. As a well-known mitochondrial targeted antioxidant, melatonin can cross the blood-brain barrier and is involved in neuronal protection [13,14], regulation of circadian rhythms [[15], [16], [17]], and antinociception [18]. Melatonin also provides an anti-inﬂammatory effect [19,20]. Emerging evidence suggested that melatonin can decrease the antinociceptive tolerance induced by morphine in mouse models [[21], [22], [23]], but the exact mechanism of the rescue action induced by this drug has not been fully understood. The lack of a scientific basis has therefore prevented any attempt to use melatonin as an efficient treatment for the alleviation of morphine-induced analgesic tolerance.

In this study, we defined the role of melatonin in morphine-induced analgesic tolerance and hypothesized that aberrant activation of NLRP3 inflammasome may contribute to morphine analgesic tolerance. We first examined the possible ameliorating effect of co-administration of melatonin with morphine on morphine-induced tolerance, then we investigated the role of NLRP3 inflammasome activation in microglia during melatonin-induced reduction of morphine-induced tolerance. We found that the NLRP3 inflammasome plays an important role in antinociceptive tolerance and melatonin pretreatment could decrease morphine-induced NLRP3 inflammasome activity. Deficiency of *Nlrp3* in mice blunted morphine-induced analgesic tolerance and acetic acid-induced pain. Our results uncovered the molecular mechanism how melatonin decreases morphine induced-analgesic tolerance.

## 2 Materials and methods

### 2.1 Reagents, cells and drug treatment

The primary antibodies and chemicals used in this study are listed in Table S1. The BV2 cells were obtained from the Kunming Cell Bank, Kunming Institute of Zoology (KIZ). Melatonin and nigericin were dissolved in ethanol. For melatonin treatment, cells were pretreated with 200 μM melatonin for 30 min before morphine or Lipopolysaccharide (LPS) treatment. LPS was dissolved in endotoxin-free water and 1 μg/mL LPS was added into medium to treat cells for 6 h. Nigericin (15 μM) was added into medium for 30 min after other drug treatment in order to further stimulate the second signal for activating NLRP3 inflammasomes [24].

### 2.2 Isolation of mouse primary microglia

Mouse primary microglia were prepared and cultured as previously described [25]. Briefly, brain cortices from 1-day old neonatal mice were dissociated with 1-mL pipettes. Debris was removed by filtration with a 70-μm cell strainer (Falcon). Cells were cultured in DMEM plus 10% fetal bovine serum (FBS, v/v) supplemented with 100 IU/mL penicillin and 100 μg/mL streptomycin. After 10 days, confluent mixed glial cultures were shaken at 37 ^o^C for 2 h to promote microglia detachment. Culture medium containing released microglia cells was aspirated, centrifuged at 1000 g for 5 min, and collected microglia were subsequently plated onto poly-D, l-ornithine-coated P100 plates for growth. Primary microglia were stained with antibody to IBA-1 (anti-mouse IBA-1, Merck Millipore, MABN92, 1:500) and had a purity >95%.

### 2.3 Heroin-addicted patients

Six female heroin addicts (mean age 31.7 ± 2.4 years) at the withdrawal and rehabilitation stages were recruited from the Kunming Rehabilitation Center on Drug Dependence in Yunnan Province, China. Six drug-free healthy volunteers (mean age 30 ± 1.7 years), without any history of medical, neurological or psychiatric diseases, were used as the control samples. All the subjects provided informed consent prior to the study. These heroin-addicted patients and controls had been described in our previous studies [21,26]. The institutional review board of Kunming Institute of Zoology approved this study (SWYX-20090302-03).

### 2.4 Animal models

The *Nlrp3*^−/−^ mouse was created using C57BL/6 as the genetic background and this mouse was previously described [27]. For all behavioral tests, C57BL/6 (as the wild-type animals: WT) and *Nlrp3*^−/−^ mice (body weight: 25–30 g) at an age of 8 weeks were used. Mice were housed in clear plastic cages with free access to water and food in an established animal house of 22 ± 2 °C and 50% humidity, with a 12-h light/dark cycle. Data acquisition and analyses were performed with a double-blind, controlled design. The related information for grouping of animals, number of animals and the treatments in this study were listed in Table S2 and Table S3. All experiments involving animals and the animal care and experimental protocol were approved by the Institutional Review Board of Kunming Institute of Zoology, Chinese Academy of Sciences (SYDW-2009017, RTSW-2009018 and SMKX-2018011).

### 2.5 Morphine analgesia and tolerance

Morphine hydrochloride was diluted in saline (0.9% NaC1). Melatonin was dissolved in saline with 0.5% ethanol (v/v), and saline with 0.5% ethanol was used as a vehicle. We chose the concentrations of melatonin (0.5 mg/kg) and morphine (10 mg/kg) that had the best effect according to our previous study [21]. Melatonin was injected intraperitoneally 30 min before subcutaneous injection of morphine [21]. Analgesia was assessed using the radiant heat tail-flick latency (Tail-Flick Unit 37360, UGO Baseline, Comerio, Italy) and a hot platform (YLS-21A, Beijing Dameida Technology Co., Ltd, China) as previously described [28]. For the tail-flick test, mice were placed in Plexiglas cages (9 × 6 × 3 cm) on a modified Hargreaves Device. Mice were habituated to the device for 2 min before each test session. A halogen lamp was focused on the tail and the withdrawal reflex time was determined by a photocell. We measured tail flick latency at IR30 and IR50 (decimal selector of heat intensity) every two days from day 0 (baseline) to day 7, 14 and 21. We used 20 s as a cutoff time to avoid damaging the tail. Baseline responses were determined for each mouse before drug injection. For morphine hot plate tolerance, repeated morphine injections (10 mg/kg subcutaneously) were given daily for 7, 14 and 21 days. The hot plate test was performed on a platform heated to 47.5 °C, 50 °C and 52.5 °C with a cutoff of 30 s, and the latency to paw lick or jump was recorded. The baseline response was determined for each animal before treatment. The analgesic response to morphine was assessed by the hot plate test or tail-flick test at 30 min or 60 min after morphine injection (10 mg/kg) [21,29]. For cross-tolerance test, the tolerance was established by repeated injections of either melatonin (or morphine) alone or the saline (or vehicle) twice a day for 2 days. On day 3, melatonin or morphine was injected to mice, followed by the tail-flick test and hot plate test as previously described [30].

### 2.6 Acetic acid-induced writhing test

Pain sensitivity was evaluated by measuring the acetic acid-induced writhing responses as previously described [31], as acetic acid could induce abdominal contractions and hind limb stretching. Mice were placed into open polyvinyl cages (20 × 40 × 15 cm) immediately after acid challenge, and abdominal constrictions were counted cumulatively over a period of 30 min.

### 2.7 Tissue preparation

Mice were anesthetized with pentobarbital at the end of the cycle of drug treatment. After having collected blood by the heart punctures, mice were intracardially perfused with ice-cold saline. Brain was extracted and split into two hemispheres. We isolated prefrontal cortex and other brain tissues from one hemisphere for protein and mRNA analyses. Tissues were frozen in liquid nitrogen and stored at −80 °C. Another hemisphere was used for immunofluorescence assay. Briefly, tissues were fixed in 4% paraformaldehyde until further processing.

### 2.8 RNA interference and transfection

siRNA oligos against *Nlrp3* (GenePharma, *Nlrp3*-Mus-384 siRNA oligo) and negative control siRNA (siNC, which was designed to have no known mRNA targets in the cells being used) were obtained from GenePharma (Suzhou, China). The BV2 cells were transfected according to the procedure in our previous studies [32,33]. In brief, cells (1 × 10^5^ per well) were seeded in 12-well plates to grow to 50% confluence. Before transfection, culture medium was removed and washed once with Opti-MEM medium (Gibco-BRL, 31985-070). The *Nlrp3* siRNA or NC siRNA was dissolved in Opti-MEM medium, and was then mixed with 3 μL Lipofectamine^TM^ 3000 (Invitrogen, L3000008) to achieve a final volume of 100 μL. The siRNA-Lipofectamine mixture was incubated at room temperature for 20 min, and added to each well together with an additional 400 μL Opti-MEM medium. The medium was removed at 6 h after transfection and fresh growth medium was added (1 mL/well) for cell growth. We optimized the siRNA concentration for transfection at a concentration of 50 nM and the time for cell harvest at 48 h.

### 2.9 Assays for reactive oxygen species (ROS) and pyroptotic cell death

The BV2 cells and primary microglia were cultured in 1640 or DMEM medium plus with 10% FBS supplemented with 100 IU/mL penicillin and 100 μg/mL streptomycin in a humidified atmosphere incubator with 5% CO_2_ at 37 °C. After morphine treatment for 6 h, cells were harvested and washed once with phosphate-buffer saline (PBS). DCFH-DA (2 μM) was used to examine intracellular ROS level. Cells were incubated with the dye at 37 °C for 20 min, then washed twice with PBS, resuspended in PBS and kept on ice for an immediate detection on the FACScan (Becton Dickinson, USA). The CytoTox 96 Non-Radioactive Cytotoxicity Assay kit (Promega) was used to assess lactate dehydrogenase (LDH) release to measure cell death according to the manufacturer's instructions.

### 2.10 Quantitative real-time PCR

Total RNA was isolated from cell using TRIZOL (Invitrogen, 15596-018). Total RNA (1 μg) was used to synthesize single-strand cDNA using the M-MLV Reverse Transcriptase (Promega, M170A) in a final volume of 25 μL according to the manufacturer’s instructions. The relative mRNA levels of *Nlrp3, IL-1β, Asc, Caspase-1, Irf7, Tlr2* and *Gsdmd* were quantified by using quantitative real-time PCR, with normalization to the *Actb/β-actin* gene. The quantitative real-time PCR was performed in a total volume of 20 μL containing 2 μL of diluted products, 10 μL of SYBR Master Mix (Takara), 0.2 uL 10 μM each primer (Table S4), on an BIO-RAD Real-time PCR detection system. The quantitative real-time PCR thermal cycling conditions were composed of a denaturation cycle at 95 °C for 5 min, followed by 40 cycles of 95 °C for 10 s and 57 °C for 30 s. For microglia single-cell quantitative real-time PCR analysis, we isolated microglial cells as previously described [34] with some modifications. In brief, adult mice were transcardially perfused with ice-cold PBS and cortex tissues were collected from control or drug-treated mice in sterile PBS containing penicillin and streptomycin (Gibco, 15140-122). The tissues were washed 5 times with PBS in clean bench, scissored into smaller pieces and enzymatically digested with collagenase IV (1 mg/mL, Thermo Fisher Scientific) and DNase I (0.2 mg/mL, Sigma). The tissues were incubated in 37 °C water bath for 15 min with gentle agitation at a 5-min interval. Single cell suspension was washed 3 times followed by filtration with 40-μm nylon cell strainer. Cell suspensions in 10 mL of cold 0.9 M sucrose solution were centrifuged at 850 g for 10 min to remove the myelin. Floating myelin and the supernatant were discarded and cell pellets were re-suspended in 15 mL centrifuge tube with 300 μL PBS contained 1% bovine serum albumin (BSA). Cells were incubated with CD11b (microglia) antibody (dilution at 1:100, Abcam, ab25533) at 4 °C for 40 min. The incubations were manually shaken at 10 min intervals. After incubation, cells were washed 2 times with PBS contained 1% BSA to remove the residual antibody, and were resuspended in DMEM contained 10% FBS for single cell picking. Single cell suspensions were checked under fluorescence microscope to ensure the microglia cells were positively stained for fluorescence. Single cell complementary DNAs (cDNAs) were prepared instantly with the method of Smart-seq2 [35,36] with some modifications. Briefly, single cells with positive fluorescence and good state were picked up with the home-made mouth pipette aided by pulled glass capillaries and washed 3 times in PBS supplemented with 0.1% BSA. Cells were lysed in lysis buffer (2 μL 0.2% Triton X-100 (Sigma), 1 μL oligo(dT) primer and 1 μL dNTP (Thermo Fisher Scientific) and full-length poly(A)-tailed RNA was reversely transcribed and amplified with 23 cycles to increase the cDNA amount. The amplification products were diluted at 1:20 with RNase-free water for the quantification of targeted genes.

### 2.11 Western blotting and ELISA

Western blot assays for the target proteins were performed using the common procedure. Cell lysates of different mouse cerebral cortex, cultured mouse primary microglia and BV2 cells were prepared as described in our previous studies [32,33]. For the supernatants, proteins were extracted using methanol-chloroform precipitation as described previously [27]. A total of 25 μg protein was separated by 12% or 15% SDS PAGE, and was transferred to a polyvinylidene difluoride membrane (Bio-Rad, L1620177 Rev D). The membrane was soaked with 5% (w/v) skim milk for 2 h at room temperature. Antibodies included those in Table S1 and peroxidase-conjugated anti-mouse (lot number 474–1806) or anti-rabbit (lot number 474–1516) IgG (1:5000; KPL). The epitope was visualized using an ECL Western blot detection kit (Millipore, WBKLS0500). ImageJ (National Institutes of Health, Bethesda, Maryland, USA) was used to evaluate the densitometry. The IL-1β levels in culture supernatants, serum from mice and heroin addicts were analyzed by using ELISA kit (R&D Systems, USA).

### 2.12 Immunofluorescence assay

Brain was sectioned coronally at 10-μm thickness on a cryostat (Leica, CM1850UV-1-1, Amtzell, Germany). Sections were collected on slides and were incubated with primary antibodies against ASC (1:500; Santa Cruz Biotechnology, sc-22514-R), ASC (1:500; Santa Cruz Biotechnology, sc-514414), CD68 (1:300; Invitrogen, MA5-13324) and TMEM119 (1:300; abcam, ab209064) for 12 h. Followed by incubation with a FITC-conjugated anti-Rabbit IgG (1:500; Invitrogen, A-21206) or anti-Mouse IgG (1:500; Invitrogen, A-11032) secondary antibody to show the target protein and with DAPI to counterstain nuclei, respectively. The slides were visualized under an Olympus FluoView™ 1000 confocal microscope (Olympus, America). We randomly selected 10 brain slices from each group and quantified with a double blind, controlled design by using ImageJ (National Institutes of Health, Bethesda, MD, USA).

### 2.13 Statistical analysis

Statistical analysis was performed by using GraphPad prism version 7.0. Sample size calculation was based on our previous study [21]. Power analysis showed that group sizes of 5–16 mice for most of behavioral tests analyzed were sufficient to detect a 20–40% improvement (ɑ = 0.05, 80% power, final sample size should be adjusted for expected attrition, see Table S2 and Table S3). The analgesic tolerance scores and acetic acid-induced writhing of WT and *Nlrp3*^−/−^ mice were assessed by using two-way repeated-measures analysis of variance (ANOVA). The data involving more than two groups were analyzed by using one-way ANOVA. Unpaired two-tailed *t*-test was used for comparing serum levels of IL-1β between healthy individuals and heroin-addicted patients. Data were represented as mean ± SEM or mean ± SD. A *P* value of <0.05 was considered to be statistically significant.

## 3 Results

### 3.1 Melatonin rescues analgesic tolerance in mice with chronic morphine treatment independent of autophagy

**Fig. 1:**
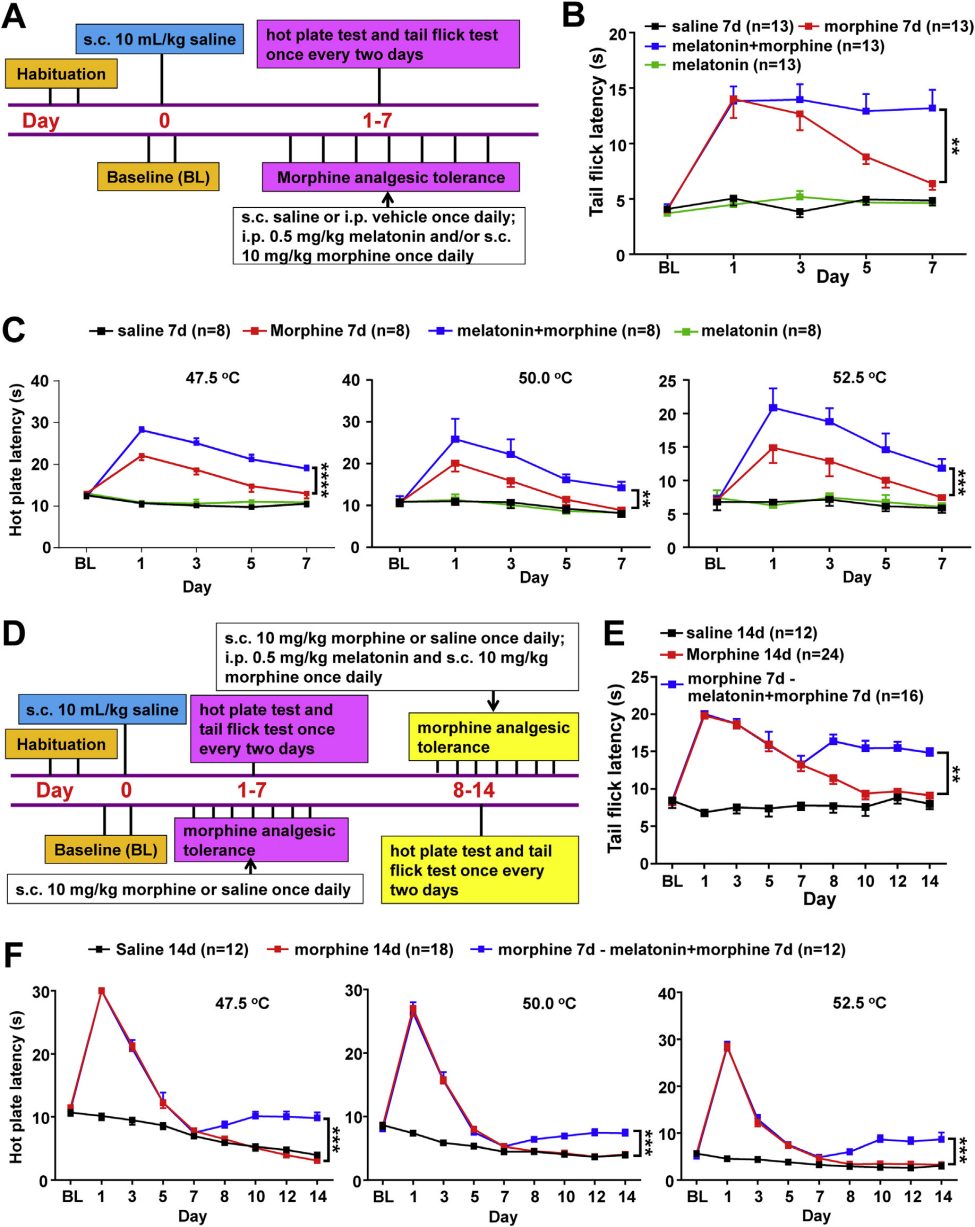
Melatonin pretreatment alleviates morphine-induced analgesia and tolerance. (*A*) A schematic profile illustrating the experimental design of the hot plate test and tail flick test with a short-term morphine treatment. (*B*) Melatonin pretreatment significantly reduced morphine-induced analgesic tolerance in the tail-flick tests with a laser density of IR30 (n = 13 animals per group. time: F_4,240_ = 21.01, *P* < 0.0001; treatment: F_3,240_ = 70.88, *P* < 0.0001; group “morphine 7d” versus group “melatonin + morphine”, 95% CI [-3.82 to -0.70], *P* = 0.0012). (*C*) Latency of paw withdrawal in the hot plate tests (n = 8 animals per group) with temperatures 47.5 °C (time: F_4,140_ = 24.41, *P* < 0.0001; treatment: F_3,140_ = 157.6, *P* < 0.0001; group “morphine 7d” versus group “melatonin + morphine”, 95% CI [-6.27 to -3.40], *P* < 0.0001), 50 °C (time: F_4,140_ = 11.49, *P* < 0.0001; treatment: F_3,140_ = 20.49, *P* < 0.0001; group “morphine 7d” versus group “melatonin + morphine”, 95% CI [-6.68 to -0.74], *P* = 0.0078), and 52.5 °C (time: F_4,140_ = 10.29, *P* < 0.0001; treatment: F_3,140_ = 32.9, *P* < 0.0001; group “morphine 7d” versus group “melatonin + morphine”, 95% CI [-6.28 to -1.67], *P* = 0.0001). (*D*) A schematic profile illustrating the experimental design of the tail flick and hotplate tests with a long-term morphine treatment. (*E-F*) Continual injection of morphine for seven consecutive days induced analgesic tolerance according to the tail flick test (*E*) (n = 12–24 animals per group. time: F_8,441_ = 20.74, *P* < 0.0001; treatment: F_2,441_ = 130.3, *P* < 0.0001; group “morphine 14d” versus group “morphine 7d-melatonin + morphine 7d”, 95% CI [-4.34 to -1.60], *P* = 0.0011) and hot plate tests (*F*) (n = 12–18 animals per group. Tests at 47.5 °C: time: F_8,230_ = 214.9, *P* < 0.0001; treatment: F_2,230_ = 133, *P* < 0.0001; group “morphine 14d” versus group “morphine 7d-melatonin + morphine 7d”, 95% CI [-2.06 to -0.55], *P* = 0.0002. Tests at 50 °C: time: F_8,296_ = 195.4, *P* < 0.0001; treatment: F_2,296_ = 104.7, *P* < 0.0001; group “morphine 14d” versus group “morphine 7d-melatonin + morphine 7d”, 95% CI [-2.16 to -0.65], *P* = 0.0007. Tests at 52.5 °C: time: F_8,257_ = 234.3, *P* < 0.0001; treatment: F_2,257_ = 225.1, *P* < 0.0001; group “morphine 14d” versus group “morphine 7d-melatonin + morphine 7d”, 95% CI [-3.54 to -1.24], *P =* 0.0005). All results are presented as mean ± SEM. Group differences were analyzed by two-way repeated-measures ANOVA. **, *P* < 0.01; ***, *P* < 0.001; ****, *P* < 0.0001.

We examined the effect of melatonin on morphine-induced analgesic tolerance in mice by using the standard hot-plate [28] and tail-flick tests [21]. Administration of morphine (10 mg/kg, subcutaneous (s.c.)) for seven consecutive days (Fig. 1A) established the antinociceptive tolerance (Fig. 1B and C). Pretreatment with melatonin (0.5 mg/kg, intraperitoneal (i.p.)) 30 min earlier than the morphine injection in mice significantly prevented the development of morphine-induced antinociceptive tolerance (Fig. 1B and C). These observations were consistent with our previous study [21] and others [37,38], and indicated that pretreatment with melatonin could prevent the development of antinociceptive tolerance induced by morphine. In mice with established analgesic tolerance (morphine injection for 7 days), co-administration of morphine and melatonin from the 8th day for 7 consecutive days (Fig. 1D) could significantly rescue the analgesic tolerance of morphine according to the tail-flick test with a laser density of IR30 (Fig. 1E) and IR50 (Fig. S1A). The salvaging effect of melatonin on morphine-induced analgesic tolerance could be replicated in mice by using the hot-plate tests at 47.5 °C, 50 °C or 52.5 °C (each with a cutoff time of 30 s), albeit the level could not be restored to the peak level (Fig. 1F). Note that melatonin still had a beneficial effect on the analgesic tolerance in mice with administration of morphine for 13 consecutive days (Fig. S2). These results suggested that melatonin could block the analgesic tolerance in mice with established analgesic tolerance.

Melatonin could salvage autophagy induced by morphine that contributed to the reduced mtDNA copy number and drug addiction [21,32]. We tested whether the beneficial effect of melatonin on morphine-induced analgesic tolerance was also mediated by autophagy, which is characterized by an increased level of MAP1LC3B-II (LC3B-II, microtubule-associated protein 1 light chain 3 beta-II) and a decreased level of SQSTM1 (p62) [39]. Consistent with our previous study [21], chronic morphine treatment elevated LC3B-II:LC3B–I ratio and decreased SQSTM1 protein level in hippocampus tissues, suggesting an increased level of autophagy (Fig. S1B). However, co-administration with melatonin from day 8 had no salvaging effect on autophagy relative to those with morphine treatment alone (Fig. S1B). Evidently, the alleviating effect of melatonin on analgesic tolerance in mice with established analgesic tolerance was independent of autophagy.

### 3.2 The effects of melatonin on chronic morphine-induced NLRP3-caspase-1 inflammasome activation

**Fig. 2:**
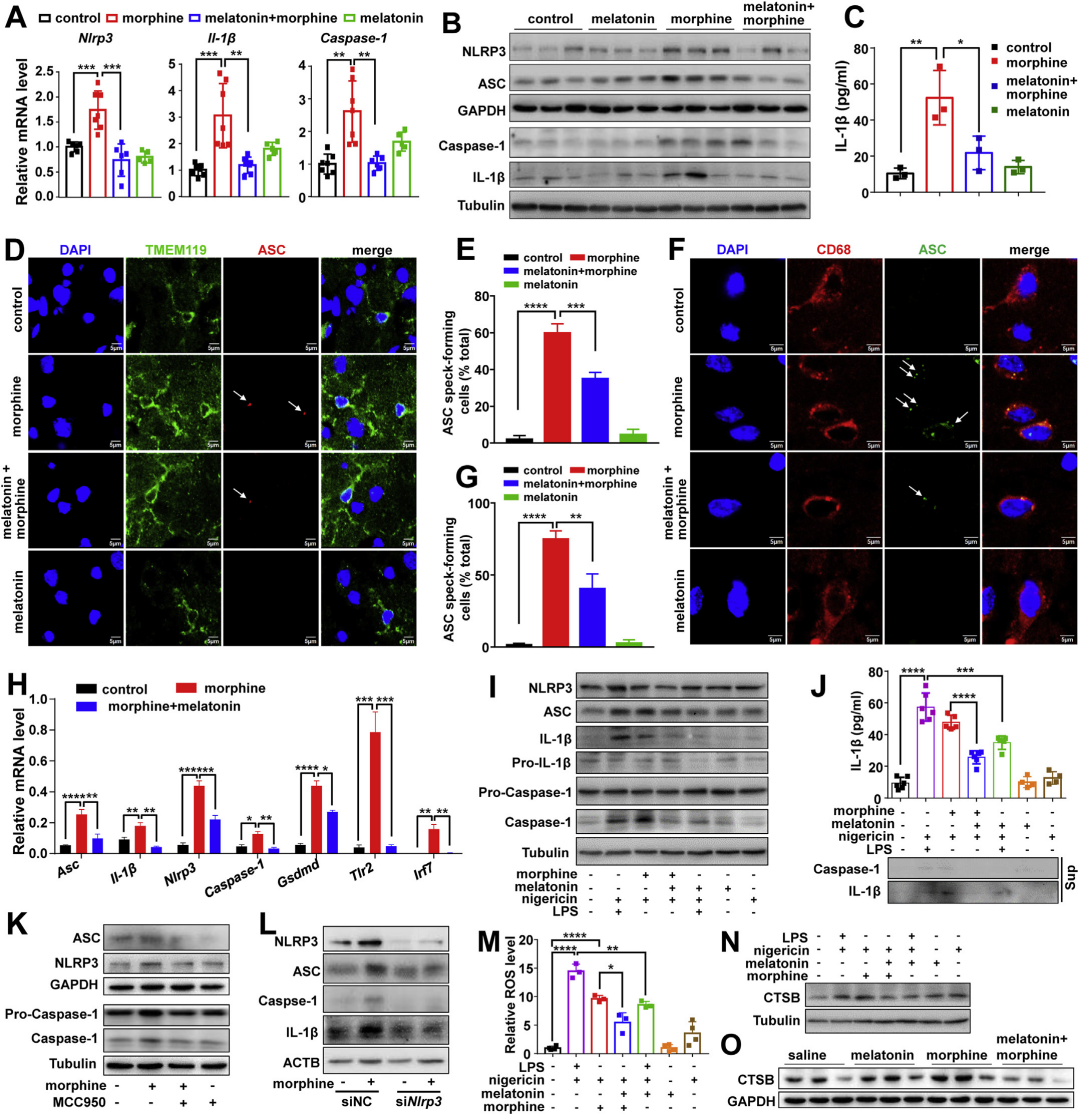
Melatonin treatment reduces morphine-induced inflammasome activation in mouse brain tissues and primary microglia and this effect is mediated by NLRP3. (*A*) mRNA levels of *Nlrp3* (n = 6–7 animals per group; F_3,20_ = 18.22, *P* < 0.0001; control group versus morphine group, 95% CI [-1.17 to -0.31], *P* = 0.0006; morphine group versus group “melatonin + morphine”, 95% CI [0.57 to 1.43], *P* = 0.0002), *Caspase-1* (n = 5–7 animals per group; F_3,21_ = 12.57, *P* < 0.0001; control group versus morphine group, 95% CI [-2.44 to -0.78], *P* = 0.0011; morphine group versus group “melatonin + morphine”, 95% CI [0.72 to 2.45], *P* = 0.0021) and *Il-1β* (n = 7 animals per group; F_3,22_ = 13.35, *P* < 0.0001; control group versus morphine group, 95% CI [-3.06 to -1.06], *P* = 0.0009; morphine group versus group “melatonin + morphine”, 95% CI [0.87 to 2.88], *P* = 0.0012) and (*B*) protein levels of NLRP3, ASC, IL-1β, Caspase-1 (p20), Pro-IL-1β and Pro-Caspase-1 in cortex tissues from mice in Fig. 1*A*. (*C*) Serum level of IL-1β in mice treated with morphine and/or melatonin (n = 3 animals per group; F_3,8_ = 12.99, *P* = 0.0019; control group versus morphine group, 95% CI [-66.01 to -17.96], *P* = 0.0023; morphine group versus group “melatonin + morphine”, 95% CI [6.58 to 54.63], *P* = 0.0150). (*D*) Double immunostaining of mouse cerebral cortex brain slices with anti-ASC (red) and anti- TMEM119 (green) antibodies to indicate NLRP3 inflammasome in microglia cells. (*E*) Percentage of microglia containing ASC foci in brain slices from animals in (*D*) (n = 10 brain slices from 3 animals per group; F_3,36_ = 59.2, *P* < 0.0001; control group versus morphine group, 95% CI [-71.38 to -44.22], *P* < 0.0001; morphine group versus group “melatonin + morphine”, 95% CI [11.32 to 38.48], *P* = 0.0001). (*F-G*) Double immunostaining of NLRP3 inflammasome in microglia cells in mouse cerebral cortex brain slices using anti-ASC (green) and anti-CD68 (red) antibodies (*F*) and quantification of microglia containing ASC foci (*G*) (n = 10 brain slices from 3 animals per group; F_3,36_ = 33.5, *P* < 0.0001; control group versus morphine group, 95% CI [-96.78 to -50.52], *P* < 0.0001; morphine group versus group “melatonin + morphine”, 95% CI [11.37 to 57.63], *P* = 0.0016). DAPI (blue) was used for nucleus staining. (*H*) mRNA levels of inflammasome-related genes in single microglia cells from cerebral cortex tissues of mice in Fig. 1*A* (n = 4–6 single cells per group. *Asc*: F_2,13_ = 18.69, *P* = 0.0001; control group versus morphine group, 95% CI [-0.26 to -0.11], *P* < 0.0001; morphine group versus group “melatonin + morphine”, 95% CI [0.07 to 0.24], *P* = 0.0014. *Il-1β*: F_2,13_ = 14.81, *P* = 0.0004; control group versus morphine group, 95% CI [-0.15 to -0.03], *P* = 0.0066; morphine group versus group “melatonin + morphine”, 95% CI [0.07 to 0.20], *P* = 0.0010. *Nlrp3*: F_2,11_ = 70.55, *P* < 0.0001; control group versus morphine group, 95% CI [-0.47 to -0.30], *P* < 0.0001; morphine group versus group “melatonin + morphine”, 95% CI [0.11 to 0.32], *P* = 0.0012. *Caspase-1*: F_2,10_ = 14.82, *P* = 0.0010; control group versus morphine group, 95% CI [-0.13 to -0.03], *P* = 0.0101; morphine group versus group “melatonin + morphine”, 95% CI [0.04 to 0.15], *P* = 0.0015. *Gsdmd*: F_2,9_ = 77.76, *P* < 0.0001; control group versus morphine group, 95% CI [-0.47 to -0.30], *P* < 0.0001; morphine group versus group “melatonin + morphine”, 95% CI [0.04 to 0.24], *P* = 0.0213. *Tlr2*: F_2,15_ = 30.63, *P* < 0.0001; control group versus morphine group, 95% CI [-1.03 to -0.46], *P* = 0.0002; morphine group versus group “melatonin + morphine”, 95% CI [0.45 to 1.02], *P* = 0.0002. *Irf7*: F_2,11_ = 17.72, *P* = 0.0004; control group versus morphine group, 95% CI [-0.24 to -0.07], *P* = 0.0010; morphine group versus group “melatonin + morphine”, 95% CI [0.07 to 0.24], *P* = 0.0012.). (*I*) Protein levels of NLRP3, ASC, Pro-IL-1β, Pro-Caspase-1, IL-1β and Caspase-1 (p20) in lysates of mouse primary microglia cells treated with morphine (200 μM) or LPS (1 μg/mL), together with or without melatonin pretreatment (200 μM) for 6 h, then stimulated with nigericin (15 μM) for 0.5 h. Cells without any treatment were included as a negative control (control). (*J*) IL-1β production (as measured by ELISA, *upper*), and protein levels of IL-1β and Caspase-1 (p20) in culture supernatants (Sup; *below*, Western blot) from primary microglia cells in (*I*) (n = 4–6 replicates per group; F_6,29_ = 71.61, *P* < 0.0001; control group versus group “LPS + nigericin”, 95% CI [-57.33 to -38.35], *P* < 0.0001; group “LPS + nigericin” versus group “LPS + melatonin + nigericin”, 95% CI [12.39 to 32.29], *P* = 0.0004; group “morphine + nigericin” versus group “morphine + melatonin + nigericin”, 95% CI [12.13 to 32.04], *P* < 0.0001). (*K*) Increased protein levels of NLRP3, ASC and Caspase-1 (p20) in primary microglia cells with morphine (200 μM) could be blocked by a pretreatment with NLRP3 inhibitor (MCC950, 100 μM). (*L*) Knockdown of *Nlrp3* by si*Nlrp3* could abolish morphine-induced protein levels of NLRP3, ASC, Caspase-1 and IL-1β in primary microglia cells. Cells were transfected with si*Nlrp3* and siNC (each 50 nM) for 48 h, then were treated with or without morphine (200 μM) for 6 h before the harvest. (*M)* Pretreatment with melatonin (200 μM) reduced the increased levels of ROS induced by morphine in primary microglia cells (n = 3–4 replicates per group; F_6,15_ = 63.43, *P* < 0.0001; control group versus group “LPS + nigericin”, 95% CI [-15.76 to -9.81], *P* < 0.0001; group “LPS + nigericin” versus group “LPS + melatonin + nigericin”, 95% CI [2.06 to 8.34], *P* = 0.0018; group “morphine + nigericin” versus group “morphine + melatonin + nigericin”, 95% CI [0.03 to 6.31], *P* = 0.047; control group versus group “morphine + nigericin”, 95% CI [-11.26 to -6.0], *P* < 0.0001). (*N–O*) Morphine exposure increased CTSB protein level in primary microglia (*N*) and mouse cortex tissues from animals in Fig. 1*A* (*O*), and this effect could be reversed by melatonin pretreatment. Data shown in (*I*, *K*–*N*) were representative of three independent experiments with similar results. Values were presented as mean ± SD in all bar graphs. Group differences were analyzed by one-way ANOVA. *, *P* < 0.05; **, *P* < 0.01; ***, *P* < 0.001; ****, *P* < 0.0001. (For interpretation of the references to colour in this figure legend, the reader is referred to the Web version of this article.)

Following the previous study showing the contribution of inflammasome activation to morphine analgesic tolerance [12] and the fact that many studies had reported anti-inflammatory properties of melatonin [20,[40], [41], [42], [43]], we investigated whether melatonin inhibits morphine analgesic tolerance via the blockade of inflammatory responses. We first analyzed the mRNA expression levels of NLRP3 inflammasome-related genes (Table S4) in prefrontal cortex (PFC) tissues from mice with chronic morphine treatment, together with or without melatonin pretreatment. Morphine treatment significantly induced the total mRNA levels of *Nlrp3*, *Il-1β* and *Caspase-1*, whereas melatonin pretreatment could abolish the induced expression of these genes by morphine (Fig. 2A). Concordantly, the NLRP3 protein level was increased in the PFC tissues from mice with morphine treatment, and this upregulation was attenuated by melatonin pretreatment (Fig. 2B). These results indicated that morphine treatment induces NLRP3 inflammasome activity and a pretreatment with melatonin could abolish this activation.

We analyzed the protein levels of the adaptor ASC (as part of NLRP3 inflammasome), Caspase-1 and IL-1β, to confirm the activated inflammasome in PFC tissues (Fig. 2B). The Caspase-1 activation was assessed by the appearance of cleaved Caspase-1 p20 or p10 [10,44,45]. Compared with the control group, the protein levels of ASC, cleaved Caspase-1 and IL-1β were increased in PFC tissues from mice with morphine treatment, whereas melatonin pretreatment before morphine injection markedly alleviated the induced ASC, cleaved Caspase-1 and IL-1β (Fig. 2B). The serum level of IL-1β was significantly lower in mice with co-administration of melatonin and morphine compared to that of mice with morphine treatment (Fig. 2C). Collectively, these results suggested that the NLRP3-CASP1 inflammasome activation was induced in mice with chronic morphine treatment, and a pretreatment of melatonin before the morphine administration had a beneficial effect.

Microglia are resident macrophages and account for 10–15% of all cells in the brain [46]. Microglia can respond to homeostatic elements by initiating an inflammatory response [47]. We tested the response of microglia to morphine with or without melatonin. We used TMEM119 as a specific marker for microglia [48] and CD68 as a marker for activated microglia that can act as a proxy for phagocytic state [49]. ASC was used to show the NLRP3 inflammasome localization and activation [9]. Morphine markedly increased the ASC specks in microglia and activated microglia cells in mouse PFC. Pretreatment with melatonin could significantly block morphine-induced ASC specks in microglia cells (Fig. 2D–G). We performed single-cell quantitative PCR for NLRP3 inflammasome-related genes in microglia to further demonstrate the responses. We found that the NLRP3 inflammasome-related genes were highly expressed in single microglia from morphine-treated mice, while melatonin pretreatment significantly reversed the NLRP3 inflammasome activation induced by morphine (Fig. 2H). Collectively, these results demonstrated that microglia are actively involved in morphine-induced NLRP3 inflammasome activation.

We used cultured primary mouse microglia and a BV2 microglial cell line to further determine the effect of morphine in activating the NLRP3 inflammasome in microglia. The protein levels of NLRP3, the adaptor ASC, cleaved Caspase-1 and mature IL-1β in primary mouse microglia were remarkably induced in response to morphine and/or LPS treatment (Fig. 2I). The levels of IL-1β and cleaved Caspase-1 (p20) in culture supernatant of morphine-treated microglia were also significantly increased compared to the untreated cells (Fig. 2J). Melatonin pretreatment markedly decreased morphine-induced NLRP3 inflammasome activation in cultured primary microglia (Fig. 2I and J). It should be noticed that melatonin pretreatment also inhibited the LPS-induced NLRP3 inflammasome activation, suggesting its general effect on ameliorating the inflammation (Fig. 2I and J). Consistently, melatonin pretreatment also blocked the morphine-induced NLRP3 inflammasome activation in mouse BV2 cells at the mRNA (Fig. S3A) and protein (Figs. S3B and C) levels.

The Caspase-1 is activated as a result of inflammasome assembly in the cytosol [50,51]. We examined the subcellular localization of Caspase-1 protein in BV2 cells by immunofluorescence. A diffused distribution of Caspase-1 staining was observed in the cytosol of untreated BV2 cells. Morphine treatment caused a dramatic morphological change of the cells and a perinuclear aggregation of Caspase-1, which could be abolished by a pretreatment with melatonin (Fig. S4A). Together, these results indicated that morphine activated the NLRP3 inflammasome in microglia, and melatonin pretreatment could block this activation.

### 3.3 Knockdown or inhibition of NLRP3 affects morphine-induced inflammasome in primary mouse microglia and BV2 cells

To define the role of NLRP3 inflammasome in response to morphine treatment, primary mouse microglia and BV2 cells were exposed to MCC950, an NLRP3 inhibitor [52], followed by morphine treatment for 6 h. Treatment of MCC950 (100 μΜ) inhibited the morphine-induced Caspase-1 processing, resulting in a reduced level of activated Caspase-1 (p20) compared to cells treated by morphine only (Fig. 2K). Similarly, the increased levels of NLRP3 and ASC by morphine were attenuated by MCC950 treatment (Fig. 2K). Primary mouse microglia cells were transfected with *Nlrp3* siRNA (si*Nlrp3*) or negative control siRNA (siNC), followed by the treatment with morphine. Knockdown of NLRP3 protein attenuated the morphine-induced Caspase-1 processing and led to a lower level of activated Caspase-1 (p20) (Fig. 2L). Similarly, NLRP3 knockdown abolished the morphine-induced upregulation of ASC and IL-1β (Fig. 2L). All these results for NLRP3 knockdown (Fig. S4B) and pharmacological inhibition by MCC950 (Fig. S4C) could be replicated in BV2 cells. Evidently, NLRP3 was involved in the morphine-induced inflammasome activation and proinflammatory cytokine (IL-1β) secretion. Knockdown or inhibition of NLRP3 could block the morphine-induced inflammasome, with a similar effect to melatonin pretreatment.

### 3.4 Melatonin alleviates morphine-induced NLRP3 inflammasome activation through blocking cathepsin B (CTSB) release and oxidative stress

Previous studies have demonstrated that the lysosomal permeabilization, release of CTSB, and generation of mitochondrial reactive oxygen species (ROS) play an important role in activating the NLRP3 inflammasome [9,53,54]. As melatonin is a well-known antioxidant that acts by eliminating cellular ROS release [20,55], we tested whether melatonin alleviates morphine-induced NLRP3 inflammasome activation by mitigating release of CTSB and ROS. Melatonin blocked the elevated levels of ROS (Fig. 2M) and CTSB (Fig. 2N) proteins induced by morphine and LPS in primary mouse microglia, and this blocking effect of melatonin could be replicated in BV2 cells (Figs. S4 D and E). Concordantly, an increased CTSB release in response to morphine treatment was observed in mouse PFC tissues, whereas melatonin pretreatment inhibited the CTSB release induced by morphine (Fig. 2O).

To examine whether the release of CTSB from lysosomes was involved in morphine-induced NLRP3 inflammasome activation, we treated BV2 cells with 10 mM NH_4_Cl to inhibit CTSB activity by moving lysosomal pH above its optimal activity [53]. Treatment with NH_4_Cl caused a reduction of the CTSB protein level and attenuated the activation of NLRP3 inflammasome (Fig. S3D). These results indicated that CTSB was critical for morphine-induced activation of the NLRP3-CASP1 inflammasome.

Activation of NLRP3 inflammasome could induce pyroptosis, a specific type of cell death characterized by cell nuclear condensation, loss of plasma membrane integrity, and late release of intracellular proteins including the cytosolic enzyme lactate dehydrogenase (LDH) [[56], [57], [58]]. We found that melatonin pretreatment significantly reduced LDH release (Fig. S3E) and inhibited the condensed nuclei (Figs. S4F and G) induced by morphine. These results demonstrated that the suppression of CTSB and ROS release is critical for the alleviating effect of melatonin on morphine-induced NLRP3-CASP1 inflammasome activation.

### 3.5 The effect of low dose of morphine (1 mg/kg) combined with melatonin in a murine pain model

**Fig. 3:**
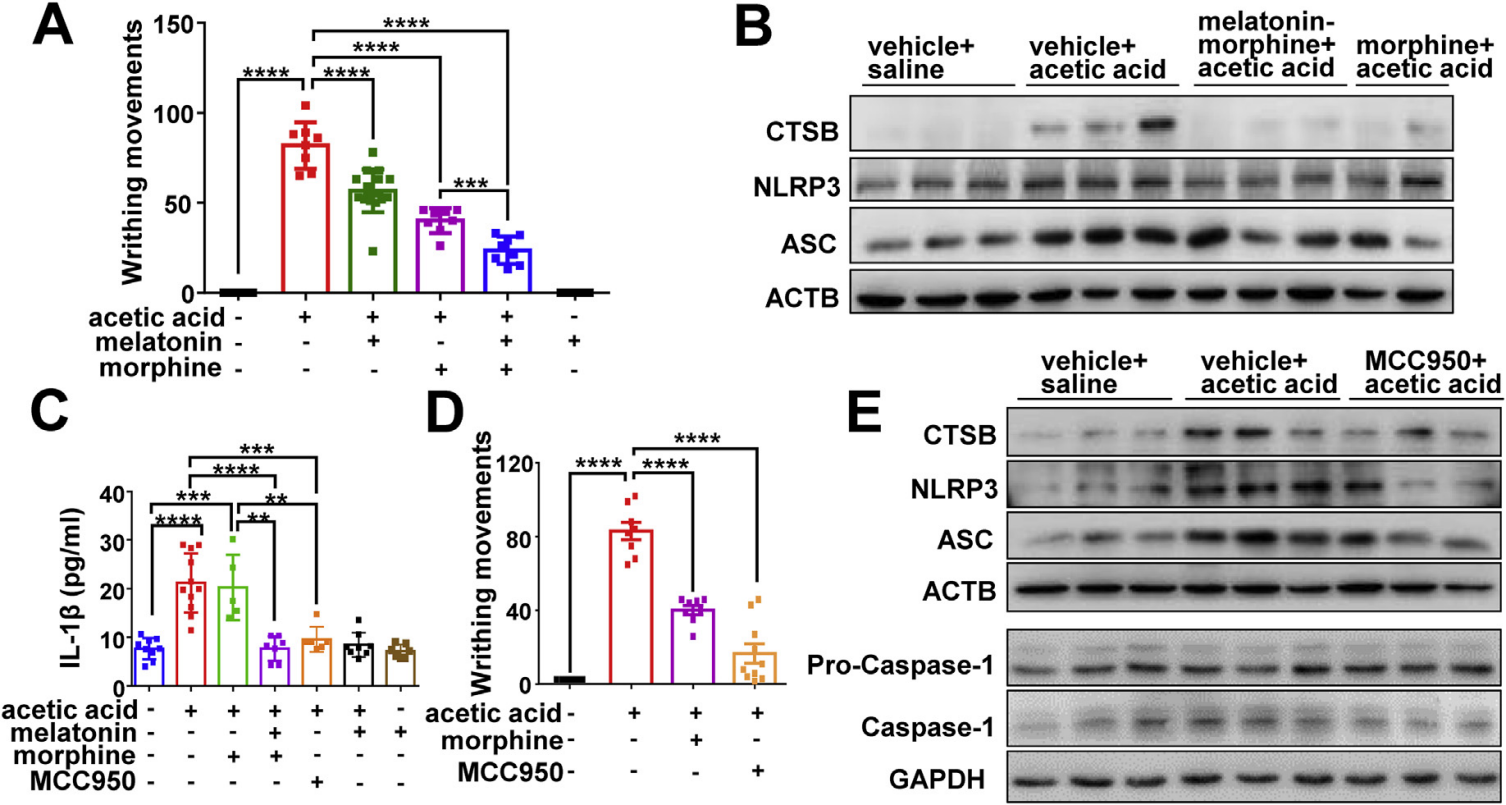
Low dose of morphine combined with melatonin has a better analgesia effect than morphine alone. (*A*) Low dose of morphine (1 mg/kg) combined with melatonin (0.5 mg/kg) has better analgesia effects than morphine alone to counteract pain induced by acetic acid (n = 8–16 animals per group; F_5,50_ = 113, *P* < 0.0001; control group versus acetic acid group, 95% CI [-95.2 to -68.55], *P* < 0.0001; acetic acid group versus group “melatonin + acetic acid”, 95% CI [13.71 to 36.79], *P* < 0.0001; acetic acid group versus group “morphine + acetic acid”, 95% CI [28.42 to 55.08], *P* < 0.0001; acetic acid group versus group “melatonin + morphine + acetic acid”, 95% CI [45.05 to 71.7], *P* < 0.0001; group “morphine + acetic acid” versus group “melatonin + morphine + acetic acid”, 95% CI:[3.30 to 29.95], *P* < 0.001). (*B*) Protein levels of CTSB, NLRP3 and ASC in mouse PFC tissues from (*A*). (*C*) Serum level of IL-1β as measured by ELISA in mice from (*A*) and (*D*) (n = 5–11 animals per group; F_6,46_ = 18.5, *P* < 0.0001; control group versus acetic acid group, 95% CI [-19.12 to -7.93], *P* < 0.0001; control group versus group “acetic acid + morphine”, 95% CI [-19.55 to -5.67], *P* = 0.0001; acetic acid group versus group “melatonin + morphine + acetic acid”, 95% CI [7.47 to 19.5], *P* < 0.0001; acetic acid group versus group “MCC950+acetic acid”, 95% CI [5.29 to 17.93], *P* = 0.0002; group “morphine + acetic acid” versus group “melatonin + morphine + acetic acid”, 95% CI [5.28 to 19.86], *P* = 0.0011; group “morphine + acetic acid” versus group “MCC950+acetic acid”, 95% CI [3.16 to 18.23], *P* = 0.0013). (*D-E*) MCC950 inhibited acetic acid-induced writhing movements (*D*) (n = 8–10 animals per group; F_3,30_ = 77.12, *P* < 0.0001; control group versus acetic acid group, 95% CI [-98.78 to -66.97], *P* < 0.0001; acetic acid group versus group “morphine + acetic acid”, 95% CI [26.85 to 58.65], *P* < 0.0001; acetic acid group versus group “MCC950+acetic acid”, 95% CI [51.09 to 81.26], *P* < 0.0001) and blocked the increased protein levels of CTSB, NLRP3, ASC and Caspase-1 (*E*). Values were presented as mean ± SD in all bar graphs. Group differences were analyzed by one-way ANOVA. **, *P* < 0.01; ***, *P* < 0.001; ****, *P* < 0.0001.

We used the acetic acid writhing model of pain to test whether melatonin combined with a low dose (1 mg/kg) of morphine could be used for relieving pain. A low dose of morphine combined with melatonin had a better analgesic effect than morphine only (Fig. 3A). Consistent with the previous study [59], we found that melatonin alone also had an analgesic effect (Fig. 3A). Analyses of the protein levels of NLRP3 inflammasome components in PFC tissues from this murine model further confirmed the beneficial effect of melatonin on counteracting the morphine-induced activation of inflammasome (Fig. 3B). Treatment of acetic acid dramatically induced IL-1β release into serum, and melatonin could completely block this effect (Fig. 3C).

The analgesia effect of melatonin on acetic-acid writhing via the NLRP3 inflammasome activation could be further demonstrated by direct inhibition of NLRP3. Compared to the acetic acid group, pretreatment of MCC950 significantly reduced the pain in mice injected with acetic acid, and the analgesic effect of MCC950 (Fig. 3D) was even comparable to that of low dose (1 mg/kg) of morphine combined with melatonin (Fig. 3A). The induced protein levels of NLRP3 and ASC (Fig. 3E), and increased IL-1β release (Fig. 3C) in the acetic acid group were attenuated by MCC950 treatment. These results supported that NLRP3 inflammasome activation plays an important role in pain [12,60], and low dose of morphine combined with melatonin, or direct delivery of NLRP3 inhibitor MCC950, might have better anti-analgesia effects for clinical usage.

### 3.6 *Nlrp3* knockout affects pain threshold and morphine-induced analgesic tolerance behavior in mice

**Fig. 4:**
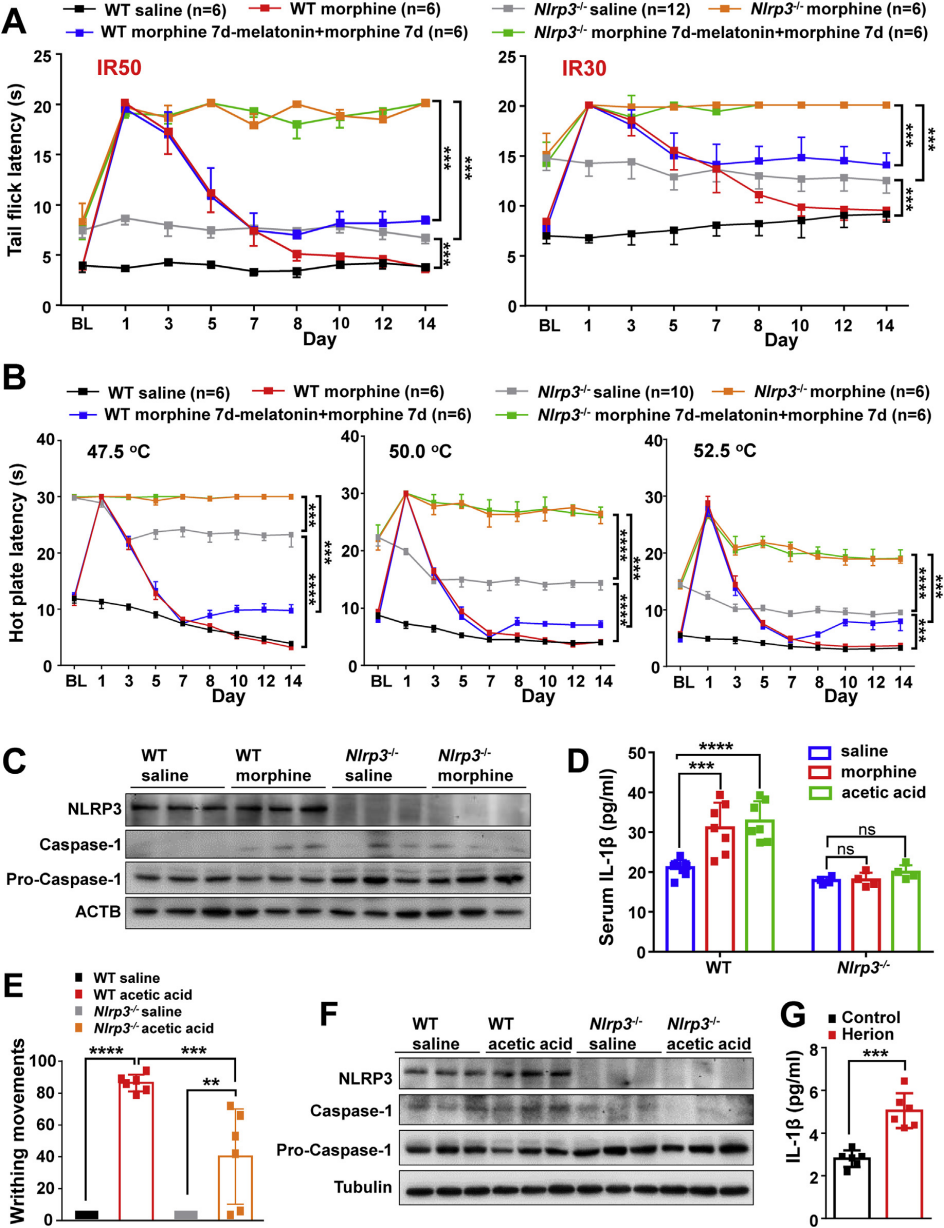
Deficiency of *Nlrp3* in mice affects morphine-induced analgesia and tolerance and blunts acetic acid-induced pain behavior. (*A-B*) Continual injection of morphine (10 mg/kg morphine, once a day for 14 days) induced analgesic tolerance according to the tail-flick tests with a laser density of IR30 or IR50 (n = 6–12 animals per group. IR30: time: F_8,324_ = 7.781, *P* < 0.0001; treatment: F_5,324_ = 82.63, *P* < 0.0001; group “*Nlrp3*^*−/−*^ morphine 7d - melatonin + morphine 7d” versus group “WT morphine 7d - melatonin + morphine 7d”, 95% CI [-6.21 to -2.32], *P* = 0.0003; *Nlrp3*^*−/−*^ morphine group versus *Nlrp3*^*−/−*^ saline group, 95% CI [-7.67 to -4.38], *P* = 0.0001; *Nlrp3*^*−/−*^ saline group versus WT saline group, 95% CI [-7.20 to -3.80], *P* = 0.0001. IR50: time: F_8,324_ = 39.62, *P* < 0.0001; treatment: F_5,324_ = 273.9, *P* < 0.0001; group “*Nlrp3*^*−/−*^ morphine 7d - melatonin + morphine 7d” versus group “WT morphine 7d - melatonin + morphine 7d”, 95% CI [-8.76 to -5.78], *P =* 0.0005; *Nlrp3*^*−/−*^ morphine group versus *Nlrp3*^*−/−*^ saline group, 95% CI [-11.62 to -9.13], *P* = 0.0002; *Nlrp3*^*−/−*^ saline group versus WT saline group, 95% CI [-5.12 to -2.54], *P =* 0.0001), and hot plate tests (n = 6–10 animals per group. Tests at 47.5 °C: time: F_8,306_ = 69.82, *P* < 0.0001; treatment: F_5,306_ = 755.2, *P* < 0.0001; group “*Nlrp3*^*−/−*^ morphine 7d - melatonin + morphine 7d” versus group “WT morphine 7d - melatonin + morphine 7d”, 95% CI [-17.41 to -14.53], *P =* 0.0001; *Nlrp3*^*−/−*^ morphine group versus *Nlrp3*^*−/−*^ saline group, 95% CI [-6.13 to -3.64], *P* = 0.0003; *Nlrp3*^*−/−*^ saline group versus WT saline group, 95% CI [-17.96 to -15.4], *P* < 0.0001. Tests at 50 °C: time: F_8,306_ = 49.5, *P* < 0.0001; treatment: F_5,306_ = 432.7, *P* < 0.0001; group “*Nlrp3*^*−/−*^ morphine 7d - melatonin + morphine 7d” versus group “WT morphine 7d - melatonin + morphine 7d”, 95% CI [-17.86 to -14.33], *P* = 0.0001; *Nlrp3*^*−/−*^ morphine group versus *Nlrp3*^*−/−*^ saline group, 95% CI [-12.25 to -9.25], *P* < 0.0001; *Nlrp3*^*−/−*^ saline group versus WT saline group, 95% CI [-12.23 to -9.24], *P* < 0.0001. Tests at 52.5 °C: time: F_8,306_ = 66.71, *P* < 0.0001; treatment: F_5,306_ = 265.2, *P* < 0.0001; group “*Nlrp3*^*−/−*^ morphine 7d - melatonin + morphine 7d” versus group “WT morphine 7d - melatonin + morphine 7d”, 95% CI [-11.47 to -8.41], *P* = 0.0001; *Nlrp3*^*−/−*^ morphine group versus *Nlrp3*^*−/−*^ saline group, 95% CI [-10.98 to -8.28], *P* < 0.0001; *Nlrp3*^*−/−*^ saline group versus WT saline group, 95% CI [-8.05 to -5.26], *P* = 0.0003) in wild type (WT) mice, but not in *Nlrp3*^−/−^ mice. The procedure was same to Fig. 1*D*. Values were presented as mean ± SEM. Group difference was analyzed by two-way repeated-measures ANOVA. ***, *P* < 0.001; ****, *P* < 0.0001. (*C*) Protein levels of NLRP3, Caspase-1 (p20) and Pro-Caspase-1 in mouse cortex tissues from (*A*). (*D*) Serum level of IL-1β in *Nlrp3*^*−/−*^ and wild-type (WT) mice as measured by ELISA (WT: n = 7–9 animals per group; F_2,20_ = 15.76, *P* < 0.0001 by one-way ANOVA; saline group versus morphine group, 95% CI [-15.81 to -4.19], *P* = 0.0009; saline group versus acetic acid group, 95% CI [-17.49 to -5.88], *P* < 0.0001. *Nlrp3*^*−/−*^: n = 4 animals per group; F_2,9_ = 2.303, *P* = 0.1557 by one-way ANOVA; saline group versus morphine group, 95% CI [-3.23 to 2.90], *P* = 0.9874; saline group versus acetic acid group, 95% CI [-5.18 to 0.95], *P* = 0.1859). (*E*) *Nlrp3* deficiency significantly reduced writhing movements compared to WT mice in the presence of acetic acid (n = 6 animals per group; Group difference was analyzed by two-way ANOVA; saline group versus acetic acid group, F_1,20_ = 104, *P* < 0.0001; WT group versus *Nlrp3*^*−/−*^ group, F_1,20_ = 13.86 *P* = 0.0013; WT saline group versus WT acetic acid group, 95% CI [-111.9 to -60.75], *P* < 0.0001; WT acetic acid group versus *Nlrp3*^*−/−*^ acetic acid group, 95% CI [20.58 to 71.75], *P* = 0.0002; *Nlrp3*^*−/−*^ saline group versus *Nlrp3*^*−/−*^ acetic acid group, 95% CI [-65.75 to -14.58], *P* = 0.0011). (*F*) Western blotting analyses for NLRP3, Caspase-1 (p20) and Pro-Caspase-1 in mouse cortex tissues from (*E*). (*G*) Serum levels of IL-1β in heroin-addicted patients (n = 6 individuals per group, t (10) = 5.376, 95% CI [1.52 to 3.68], *P* = 0.0003 by unpaired two-tailed *t*-test). Bar graphs (mean ± SD) in (*D-E*) and (*G*) refer to the result of all samples. **, *P* < 0.01; ***, *P* < 0.001; ****, *P* < 0.0001; ns, not significant.

The alleviating effect of melatonin on morphine-induced analgesia tolerance via the mediation of NLRP3 inflammasome activation could be demonstrated by using *Nlrp3*^***−/−***^ mice. As described before (Fig. 1), repeated morphine administration in WT mice over 7 days resulted in an analgesic tolerance at a laser density of IR30 and IR50 (Fig. 4A) and three different temperatures of hot-plates (Fig. 4B). However, the *Nlrp3*^***−/−***^ mice did not develop morphine analgesic tolerance in these models (Fig. 4A and B). Accordingly, melatonin treatment before morphine administration had no effect on morphine-induced analgesic tolerance in *Nlrp3*^***−/−***^ mice (Fig. 4A and B), suggesting that the expected beneficial effect of melatonin was mediated by NLRP3. There was no NLRP3 inflammasome activation in PFC tissues of *Nlrp3*^***−/−***^ mice, regardless of morphine treatment (Fig. 4C). We failed to detect the IL-1β release in serum of *Nlrp3*^***−/−***^ mice with or without morphine treatment (Fig. 4D). These findings further showed the active involvement of NLRP3 inflammasome activation in the development of morphine-induced analgesic tolerance.

We assessed the pain in *Nlrp3*^*−/−*^ mice injected with or without acetic acid. The WT and *Nlrp3*^*−/−*^ mice with saline injection had no apparent writhing movements (Fig. 4E). Injection of acetic acid in WT mice resulted in a significant increase of writhing movements relative to the saline controls (Figs. 3A and 4E). *Nlrp3* deficiency significantly reduced the number of writhing movements compared to WT mice injected with acetic acid (Fig. 4E), although *Nlrp3*^*−/−*^ mice also presented writhing movements in response to acetic acid. The induced levels of NLRP3 and Caspase-1 (p20) (Fig. 4F) and IL-1β release (Fig. 4D) by acetic acid were attenuated in *Nlrp3*^*−/−*^ mice. These results based on *Nlrp3*^*−/−*^ mice showed that the NLRP3 inflammasome was actively involved in morphine-induced analgesic and affected pain response.

### 3.7 Opiate-addicted patients have an increased level of serum IL-1β

Opiate-addicted patients often have opioid-induced hyperalgesia [61,62]. We previously showed that heroin-addicted patients had a significantly lower level of serum melatonin compared to healthy individuals [21], and this might be associated with the pain sensitivity in these patients based on the above results. We found that the level of serum IL-1β in heroin-addicted patients was significantly higher than that of healthy individuals (Fig. 4G). The decreased melatonin in opiate-addicted patients would be associated with an increase of NLRP3 inflammasome, which in turn led to pain sensitivity. It could therefore be useful to use melatonin as an adjunct to relieve the pain of opiate-addicted patients, and a clinical trial could be performed to confirm this practice.

## 4 Discussion

**Fig. 5:**
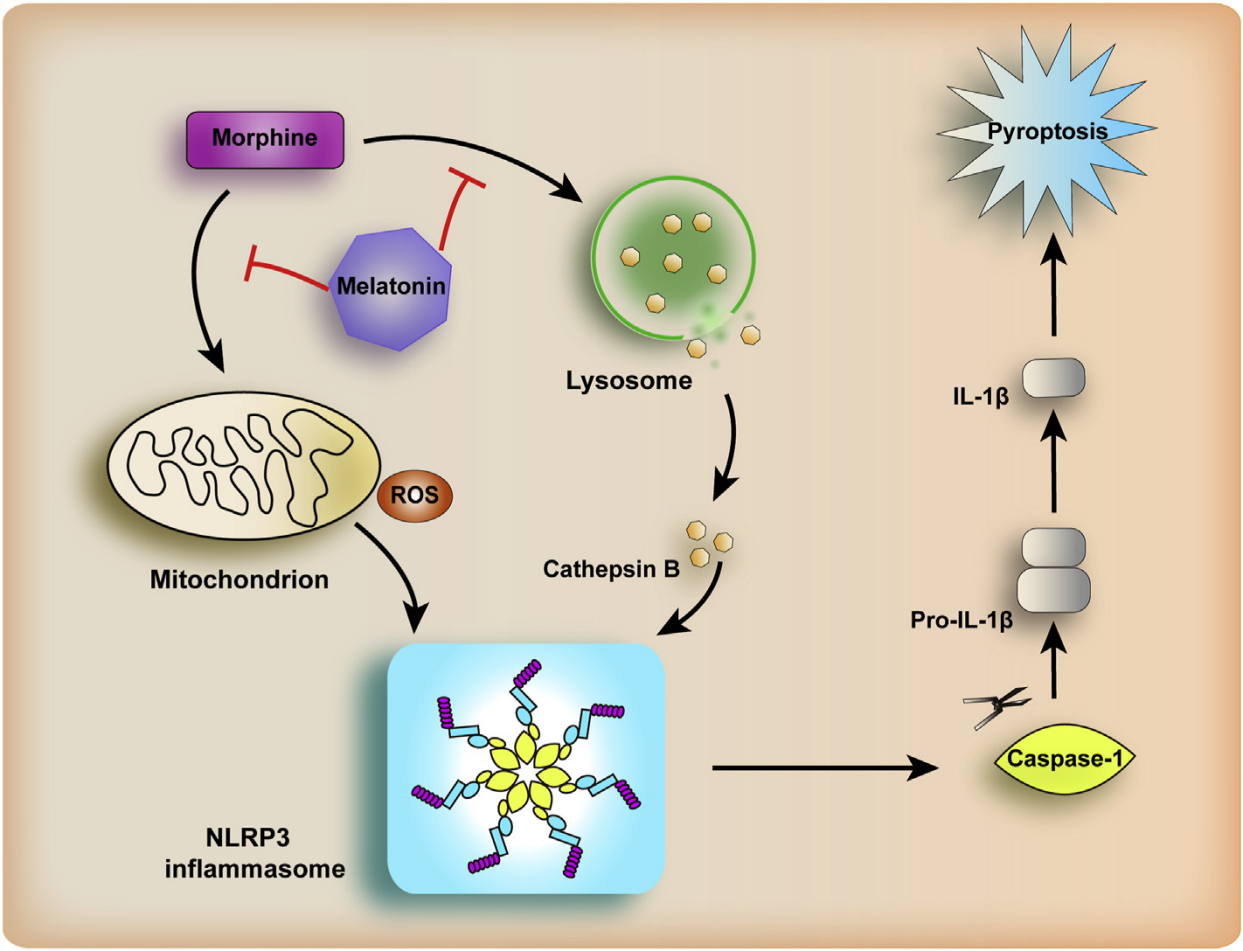
A proposed mechanism of pretreatment with melatonin in alleviating morphine-induced analgesic tolerance. Melatonin decreased NLRP3 inflammasome activation induced by morphine.

Morphine has been widely used to relieve pain, but it causes analgesic tolerance and hyperalgesia [3,63]. It is important to find a way to avoid the side effects of morphine and related opioids. In this study, we have provided several lines of evidence to show that melatonin could alleviate the side effects of morphine by targeting the NLRP3 signaling in microglia and relieved morphine-induced analgesic tolerance. We have shown the potential mechanism by which chronic morphine exposure leads to excess production of cellular ROS and activation of NLRP3 inflammasome in microglia. Melatonin could diminish ROS and consequently inhibited the activation of NLRP3 inflammasome, suppressed the over-activated IL-1β signaling, which finally attenuated the development of morphine analgesic tolerance (Fig. 5). This effect was independent of autophagy, which has been shown to be involved in drug addiction [21,64,65] and presented an *Atg5*-and *Atg7*-dependent, dopaminergic neuron specific pattern [32]. These findings indicated that melatonin enacted different roles in counteracting morphine-induced antinociceptive tolerance compared to its salvaging effect on autophagy to prevent morphine-induced behavioral sensitization [21]. The diverse effects of melatonin might be caused by its multiple functions in morphine addiction and analgesic tolerance. There was no cross-tolerance between melatonin and morphine, as we found that repeated injections of melatonin twice daily for 2 days did not affect morphine potency on day 3 compared with vehicle-pretreated controls (Fig. S5A). Similarly, pretreatment with morphine had no effect on melatonin potency (Fig. S5B). Our results offered new insights into the cellular events underlying chronic morphine analgesic tolerance and indicated melatonin as a promising drug for the optimization of the analgesic actions of morphine. Since melatonin pretreatment also blocked NLRP3 inflammasome activation following LPS treatment (Fig. 2I and J), it would be rewarding to test whether melatonin functioned in a more general mechanism than just blocking morphine induction, and whether it had a similar effect on alleviating the detrimental side effects of other opioid analgesics.

Recently, accumulating evidence indicated that inflammatory responses play an important role in morphine-induced antinociceptive tolerance [12,66]. Chronic morphine treatment increased glia activation in the spinal cord and posterior cingulate cortex [67], which might release proinflammatory cytokines and other substances to enhance pain. We found that the NLRP3 inflammasome was widely activated in mouse PFC tissues upon morphine treatment, whereas melatonin pretreatment decreased this effect. Studies performed in BV2 cells and primary microglia cells, and immunostaining for microglia in brain PFC tissues demonstrated that morphine-induced NLRP3 inflammasome could be abolished by melatonin pretreatment. Knockdown of *Nlrp3* reduced the elevated protein levels of ASC, activated Caspase-1 (p20) and the release of IL-1β in response to morphine treatment. Consistent with previous studies for the induced cell pyroptosis by Caspase-1 activation [57,58,68], melatonin could also block morphine-induced BV2 cell pyroptosis by reducing inflammasome activation. The observation of a better anti-analgesia effect in response to co-administration of melatonin and a low dose of morphine in mouse pain models suggested the potential usage of melatonin in the treatment of pain requiring morphine.

The active involvement of NLRP3 in morphine analgesic tolerance could be further confirmed using both pharmacological inhibition (inhibitor MCC950 [52]) and knockdown (*Nlrp3* siRNA) approaches. We demonstrated that MCC950 treatment abolished NLRP3 inflammasome activation and IL-1β release in microglia treated with morphine. Similarly, knockdown of NLRP3 in microglia cells and deficiency of *Nlrp3* in mice reduced morphine-induced downstream effectors Caspase-1 and IL-1β. Treatment of MCC950 and genetic knockout of *Nlrp3* relieved acetic acid-induced pain in mouse model. As mitochondria and ROS are critical for NLRP3 inflammasome activation [69,70], we showed that melatonin blocked morphine-induced NLRP3 inflammasome *in vitro* and *in vivo* by decreasing the generation of ROS level and CTSB release, which were reported to activate NLRP3 [53,54,66,71,72] and suppress morphine antinociceptive tolerance [73].

The observation that morphine-induced antinociceptive tolerance could be fully abolished by melatonin pretreatment or MCC950 has provided the basis for initiating a clinical trial for the usage of melatonin together with a low dose of morphine, or simply the use of MCC950 to relieve pain. The increased serum IL-1β level in heroin-addicted patients compared to healthy individuals was in agreement with the expected result of decreased melatonin in these patients [21] considering the alleviating effect of melatonin. This suggested that alleviation of NLRP3 inflammasome could be a target for counteracting morphine analgesic tolerance. Whether other pathways might be also involved in this alleviating process by melatonin, such as the canonical pathway of ion channels [[74], [75], [76]], the peripheral GABAergic system [77], the protein kinase C and N-methyl-d-aspartate receptors activity [37], microglia activation and HSP27 expression [38], awaits further study. Note that different dosages of melatonin were required to achieve the same antinociception effect in the tail-flick and hot-plate tests (Fig. 1) and in the acetic acid writhing test (Fig. 3), which were used to test acute thermal pain [78] and acute visceral pain [79], respectively. The underlying molecular mechanisms/circuits of these two types of pain are different, and we believe this is the reason why different dosages of melatonin were needed. Therefore, optimization of melatonin concentrations should be considered to counteract morphine-induced analgesic tolerance for different types of pain.

In summary, we found that chronic morphine treatment caused a release of ROS and CTSB, which led to an excessive activation of NLRP3 inflammasome activity and pyroptosis in microglia. The detrimental effect of morphine could be counteracted by melatonin treatment or NLRP3 inhibition, which prevented the development of morphine analgesic tolerance (Fig. 5). Importantly, a low-dose morphine combined with melatonin had better analgesia effects in murine pain models. Our results provided a possible new solution to improve the analgesic efficacy of morphine by the inhibition of microglial NLRP3 inflammasome. As melatonin was found to be safe and effective for inhibiting morphine tolerance, it may be a drug candidate to reduce morphine analgesic tolerance and to enhance the clinical usefulness of morphine and its related opioids.

## Disclosure of potential conflicts of interest

There were no potential conflicts of interest to be disclosed.

## Author contributions

Yong-Gang Yao, Qianjin Liu and Ling-Yan Su conceived and designed the experiments. Qianjin Liu, Ling-Yan Su, Chunli Sun, Lijin Jiao, Ying Miao, Min Xu, Rongcan Luo and Xin Zuo performed the experiments and analyzed the data. Ping Zheng, Tian Xue and Wei Xiong contributed with interpretation of the results. Rongbin Zhou offered key reagents. Yong-Gang Yao, Qianjin Liu and Ling-Yan Su wrote the manuscript. All authors reviewed the content and approved the final version for publication.
