# Supplementary material for: Melatonin alleviates morphine analgesic tolerance in mice by decreasing NLRP3 inflammasome activation
Source: Redox Biol. 2020 Apr 29;34:101560. doi: 10.1016/j.redox.2020.101560 (PMC7225735; doi:10.1016/j.redox.2020.101560)
Supplement: Supplementary data [file mmc1.docx]

**Supplementary Information**

**Table S1.** Primary antibodies and chemicals used in this study

| **Primary antibody** | **Source** | **Catalog no.** | **Western blot** |
| --- | --- | --- | --- |
| Rabbit monoclonal anti-NLRP3 | Cell Signaling Technology | D4D8T | 1:1000 |
| Mouse monoclonal anti-Caspase-1 | Adipogen | AG-20B-0042 | 1:1000 |
| Rabbit monoclonal anti-IL-1beta | Abcam | ab82558 | 1:1000 |
| Rabbit monoclonal anti-CTSB | ABZOOM | AM2528 | 1:1000 |
| Rabbit polyclonal anti-ASC | Santa Cruz Biotechnology | sc-22514-R | 1:1000 |
| Mouse monoclonal anti-Tubulin | EnoGene | E1C601 | 1:1000 |
| Mouse monoclonal anti-GAPDH | Proteintech | 60004-1-Ig | 1:10000 |
| Mouse monoclonal anti-ACTB | Beijing Zhong Shan-Golden Bridge Biological Technology CO., LTD | TA-09 | 1:10000 |
| **Chemical** | **Source** | **Catalog no.** |  |
| Melatonin | Sigma | M5250 | - |
| LPS | Sigma | L2630 | - |
| Nigericin | Merck Millipore | 481990 | - |
| NH_4_Cl | Sigma | A9434 | - |
| Morphine hydrochloride | Shenyang Pharmaceutical Co. Ltd | H21022436 | - |

**Table S2.** Grouping of animals, number of mice and their treatments for morphine analgesia and tolerance test (i.p, intraperitoneally; s.c, subcutaneously)

| Group | i.p injection | s.c injection | Test | Figure | Number |
| --- | --- | --- | --- | --- | --- |
| saline 7d | vehicle | saline | tail-flick test IR30 | Figure 1B | n=13 |
|  |  |  | hot-plate test | Figure 1C | n=8 |
| morphine 7d | vehicle | morphine | tail-flick test IR30 | Figure 1B | n=13 |
|  |  |  | hot-plate test | Figure 1C | n=8 |
| melatonin + morphine 7d | melatonin | morphine | tail-flick test IR30 | Figure 1B | n=13 |
|  |  |  | hot-plate test | Figure 1C | n=8 |
| melatonin 7d | melatonin | saline | tail-flick test IR30 | Figure 1B | n=13 |
|  |  |  | hot-plate test | Figure 1C | n=8 |
| saline 14d | vehicle | saline | tail-flick test IR30/50 | Figure 1E and S1A | n=12 |
|  |  |  | hot-plate test | Figure 1F | n=12 |
| morphine 14d | vehicle | morphine | tail-flick test IR30/50 | Figure 1E and S1A | n=24 |
|  |  |  | hot-plate test | Figure 1F | n=18 |
| morphine 7d –  melatonin + morphine 7d | vehicle 1-7d  melatonin 8-14d | morphine 1-7d  morphine 8-14d | tail-flick test IR30/50 | Figure 1E and S1A | n=16 |
|  |  |  | hot-plate test | Figure 1F | n=12 |
| saline 21d | vehicle | saline | tail-flick test IR30/50 | Figure S2B | n=8 |
|  |  |  | hot-plate test | Figure S2C | n=6 |
| morphine 21d | vehicle | morphine | tail-flick test IR30/50 | Figure S2B | n=8 |
|  |  |  | hot-plate test | Figure S2C | n=6 |
| morphine 13d –  melatonin + morphine 8d | vehicle | morphine | tail-flick test IR30/50 | Figure S2B | n=8 |
|  |  |  | hot-plate test | Figure S2C | n=6 |
| WT saline 14d | vehicle | saline | tail-flick test IR30/50 | Figure 4A | n=6 |
|  |  |  | hot-plate test | Figure 4B | n=6 |
| WT morphine 14d | vehicle | morphine | tail-flick test IR30/50 | Figure 4A | n=6 |
|  |  |  | hot-plate test | Figure 4B | n=6 |
| WT morphine 7d -melatonin + morphine 7d | vehicle 1-7d  melatonin 8-14d | morphine 1-7d  morphine 8-14d | tail-flick test IR30/50 | Figure 4A | n=6 |
|  |  |  | hot-plate test | Figure 4B | n=6 |
| *Nlrp3*^-/-^ saline 14d | vehicle | saline | tail-flick test IR30/50 | Figure 4A | n=12 |
|  |  |  | hot-plate test | Figure 4B | n=10 |
| *Nlrp3*^-/-^ morphine 14d | vehicle | morphine | tail-flick test IR30/50 | Figure 4A | n=6 |
|  |  |  | hot-plate test | Figure 4B | n=6 |
| *Nlrp3*^-/-^ morphine 7d -melatonin + morphine 7d | vehicle 1-7d  melatonin 8-14d | morphine 1-7d  morphine 8-14d | tail-flick test IR30/50 | Figure 4A | n=6 |
|  |  |  | hot-plate test | Figure 4B | n=6 |
| Group | Day 1-2 | Day 3 | **Test** | **Figure** | **Number** |
| vehicle 2d -morphine | vehicle | morphine | tail-flick test IR50  hot plate test 52.5°C | Figure S5 | n=5 |
| melatonin 2d -morphine | melatonin | morphine |  |  | n=5 |
| saline 2d -melatonin | saline | melatonin |  |  | n=5 |
| morphine 2d -melatonin | morphine | melatonin |  |  | n=5 |

**Table S3.** Grouping of animals, number of mice and their treatments for acetic acid-induced writhing test

| Group | i.p injection | i.p injection | Figure | Number |
| --- | --- | --- | --- | --- |
| vehicle + saline group | vehicle | saline | Figure 3A | n=8 |
|  |  |  | Figure 3D | n=8 |
| vehicle + acetic acid | vehicle | acetic acid | Figure 3A | n=8 |
|  |  |  | Figure 3D | n=8 |
| morphine + acetic acid | morphine | acetic acid | Figure 3A and 3D | n=8 |
| melatonin + acetic acid | melatonin | acetic acid | Figure 3A | n=16 |
| melatonin + morphine -acetic acid | melatonin +morphine | acetic acid | Figure 3A | n=8 |
| melatonin group | melatonin | saline | Figure 3A | n=8 |
| MCC950 + acetic acid | MCC950 | acetic acid | Figure 3D | n=8 |
| WT vehicle + saline | vehicle | saline | Figure 4E | n=6 |
| WT vehicle + acetic acid | vehicle | acetic acid | Figure 4E | n=6 |
| *Nlrp3*^-/-^ vehicle + saline | vehicle | saline | Figure 4E | n=6 |
| *Nlrp3*^-/-^ vehicle + acetic acid | vehicle | acetic acid | Figure 4E | n=6 |

**Table S4.** Primer pairs for detecting mRNA levels of the targeted genes in mouse tissues and single microglia cells

| **Primer** | **Sequence (5’-3’)** | **Product length (bp)** |
| --- | --- | --- |
| *Nlrp3* Forward | ATTACCCGCCCGAGAAAGG | 141 |
| *Nlrp3* Reverse | TCGCAGCAAAGATCCACACAG |  |
| *Caspase-1* Forward | CCAGAGCACAAGACTTCTGAC | 339 |
| *Caspase-1* Reverse | TGGTGTTGAAGAGCAGAAAGC |  |
| *Il-1β* Forward | GAAATGCCACCTTTTGACAGTG | 116 |
| *Il-1β* Reverse | TGGATGCTCTCATCAGGACAG |  |
| *Asc* Forward | GACAGTGCAACTGCGAGAAG | 106 |
| *Asc* Reverse | CGACTCCAGATAGTAGCTGACAA |  |
| *Irf7* Forward | CCCCAGCCGGTGATCTTTC | 125 |
| *Irf7* Reverse | CACAGTGACGGTCCTCGAAG |  |
| *Tlr2* Forward | CACCACTGCCCGTAGATGAAG | 148 |
| *Tlr2* Reverse | AGGGTACAGTCGTCGAACTCT |  |
| *Gsdmd* Forward | TTCCAGTGCCTCCATGAATGT | 193 |
| *Gsdmd* Reverse | GCTGTGGACCTCAGTGATCT |  |
| *Actin* Forward | GATGGTGGGAATGGGTCAGA | 120 |
| *Actin* Reverse | TCCATGTCGTCCCAGTTGGT |  |

**Figure S1.** **Pretreatment with melatonin alleviated morphine analgesic tolerance in a mouse model, and this effect** **was independent on autophagy.** (*A*) Continual injection of morphine for 7 days induced analgesic tolerance according to the tail-flick test with a laser density of IR50. From day 8, melatonin pretreatment before morphine administration for another 7 days significantly blocked the morphine-induced analgesic tolerance (n = 12-24 animals per group. time: F_8,441_ = 40.63, *P* < 0.0001; treatment: F_2,441_ = 109.88, *P* < 0.0001 by two-way repeated-measures ANOVA; morphine14 d group versus group “morphine 7 d - melatonin+morphine 7 d”, 95% CI [-2.33 to -0.47], *P* = 0.0013). (*B*) Pretreatment of melatonin had no effect on morphine-induced autophagy in the hippocampus tissues of mice with morphine treatment in (*A*). The result is presented as mean ± SEM. **, *P*<0.01.

**Figure S2.** **Pretreatment with melatonin ameliorated morphine analgesic tolerance in established tolerance mouse model.** (*A*) A schematic profile illustrating the experimental design of the tail-flick and hot-plate tests in mice with analgesic tolerance. (*B* and *C*) Melatonin alleviated morphine-induced analgesic tolerance in established tolerance mouse model. Continual injection of morphine for 13 days induced analgesic tolerance according to the tail-flick test (*B*) (n = 8 animals per group. IR30: time: F_11,252_ = 18.4, *P* < 0.0001; treatment: F_2,252_ = 87.58, *P* < 0.0001; group “morphine 21d” versus group “morphine13 d – melatonin+morphine 8 d”, 95% CI [-2.23 to -0.21], *P* = 0.0004. IR50: time: F_11,252_ = 18.4, *P* < 0.0001; treatment: F_2,252_ = 87.58, *P* < 0.0001; group “morphine 21 d” versus group “morphine13 d – melatonin+morphine 8 d”, 95% CI [-2.15 to -0.24], *P* = 0.0009) and hot plate test (*C*) (n = 6 animals per group. Tests at 47.5^o^C: time: F_11,180_ = 340.5, *P* < 0.0001; treatment: F_2,180_ = 105.3, *P* < 0.0001; group “morphine 21 d” versus group “morphine13 d – melatonin+morphine 8 d”, 95% CI [-2.09 to -0.90], *P* = 0.0001. Tests at 50^o^C: time: F_11,180_ = 365, *P* < 0.0001; treatment: F_2,180_ = 182.2, *P* < 0.0001; group “morphine 21 d” versus group “morphine13 d – melatonin+morphine 8 d”, 95% CI [-1.29 to -0.32], *P* = 0.0004. Tests at 52.5^o^C: time: F_11,180_ = 174.8, *P* < 0.0001; treatment: F_2,180_ = 131.3, *P* < 0.0001; group “morphine 21 d” versus group “morphine13 d – melatonin+morphine 8 d”, 95% CI [-1.66 to -0.45], *P* = 0.0001), and melatonin pretreatment from day 14 before morphine administration for 8 consecutive days significantly ameliorated morphine-induced analgesic tolerance. All results are presented as mean ± SEM. Group differences were analyzed by two-way repeated-measures ANOVA. ***, *P*<0.001.

**Figure S3. Melatonin pretreatment alleviated morphine-induced NLRP3 inflammasome activation in BV2 cells.** The BV2 cells were cultured in 1640 medium supplemented with 10% FBS and were treated with morphine (200 μM) or LPS (1 μg/mL) with or without melatonin (200 μM) for 6 h, then were stimulated with nigericin (15 μM) for 0.5 h. (*A*) The mRNA levels of *Nlrp3*, *Asc*, *Caspase-1* and *Il-1β* in BV2 cells were detected by using quantitative real-time PCR (n = 3 replicates per group. *Nlrp3*: F_4,10_ = 16.29, *P* = 0.0002; control group versus group “LPS+nigericin”, 95% CI [-0.79 to -0.03], *P* = 0.0108; group “LPS+nigericin” versus group “LPS+melatonin+nigericin”, 95% CI [0.13 to 0.89], *P* = 0.0447; group “morphine+nigericin” versus group “morphine+melatonin+nigericin”, 95% CI [0.12 to 0.89], *P* = 0.0042. *Asc*: F_4,10_ = 19.09, *P* = 0.0001; control group versus group “LPS+nigericin”, 95% CI [-0.69 to -0.13], *P* = 0.001; group “LPS+nigericin” versus group “LPS+melatonin+nigericin”, 95% CI [0.18 to 0.65], *P* = 0.0012; group “morphine+nigericin” versus group “morphine+melatonin+nigericin”, 95% CI [0.11 to 0.58], *P* = 0.0043. *Caspase-1*: F_4,10_ = 12.47, *P* = 0.0007; control group versus group “LPS+nigericin”, 95% CI [-0.55 to -0.06], *P* = 0.0148; group “LPS+nigericin” versus group “LPS+melatonin+nigericin”, 95% CI [0.12 to 0.61], *P* = 0.0042; group “morphine+nigericin” versus group “morphine+melatonin+nigericin”, 95% CI [0.01 to 0.51], *P* = 0.0359. *Il-1β*: F_4,10_ = 43.61, *P* < 0.0001; control group versus group “LPS+nigericin”, 95% CI [-12.57 to -6.64], *P* = 0.0012; group “LPS+nigericin” versus group “LPS+melatonin+nigericin”, 95% CI [1.27 to 7.21], *P* = 0.0216; group “morphine+nigericin” versus group “morphine+melatonin+nigericin”, 95% CI [-8.29 to -2.35], *P* = 0.0091). (*B*) The IL-1β protein level in culture supernatant of BV2 cells measured by ELISA (n = 3-5 replicates per group. F_4,16_ = 11.89, *P* = 0.0001; control group versus group “LPS+nigericin”, 95% CI [-49.59 to -8.64], *P* = 0.0018; group “LPS+nigericin” versus group “LPS+melatonin+nigericin”, 95% CI [2.83 to 43.79], *P* = 0.0219; group “morphine+nigericin” versus group “morphine+melatonin+nigericin”, 95% CI [1.05 to 45.63], *P* = 0.0479). (*C*) The protein levels of NLRP3, ASC, Pro-IL-1β, Pro-caspase-1, IL-1β and Caspase-1 (p20) in cell lysate were determined by Western blot. (*D*) Pretreatment with NH_4_Cl (10 mM) blocked the morphine-induced levels of CTSB and NLRP3-CASP1 inflammasome in BV2 cells. (*E*) The LDH release induced by morphine could be mitigated by melatonin pretreatment (n = 3-5 replicates per group. F_6,26_ = 12.87, *P* < 0.0001; control group versus group “LPS+nigericin”, 95% CI [-46.82 to -9.06], *P* = 0.0012; control group versus group “morphine+nigericin”, 95% CI [-30.32 to -7.30], *P* = 0.0024; group “LPS+nigericin” versus group “LPS+melatonin+nigericin”, 95% CI [4.12 to 43.26], *P* = 0.0104; group “morphine+nigericin” versus group “morphine+melatonin+nigericin”, 95% CI [8.71 to 31.43], *P* = 0.0012). Data shown in were representative of three independent experiments with similar results. Values in (*A*, *B* and *E*) were presented as mean ± SD. Group differences were analyzed by one-way ANOVA. *, *P*<0.05; **, *P*<0.01.

**Figure S4. Melatonin pretreatment reversed the morphine-induced ROS, CTSB and pyroptosis in BV2 cells.** (*A*) Subcellular distribution of Caspase-1 in BV2 cells treated with LPS, morphine, together with or without melatonin. (*B-C*) NLRP3 knockdown by si*Nlrp3* (50 nM; *B*) or pretreatment with MCC950 (100 μM; *C*) attenuated the increased protein levels of NLRP3, ASC and Caspase-1. (*D-E*) Pretreatment with melatonin (200 μM) reduced the increased CTSB protein (*D*) and ROS (*E*) levels (n = 3 replicates per group. F_4,10_ = 51.27, *P* < 0.0001; control group versus group “LPS+nigericin”, 95% CI [-5.80 to -1.87], *P* = 0.0032; control group versus group “morphine+nigericin”, 95% CI [-5.03 to -1.10], *P* = 0.0051; group “LPS+nigericin” versus group “LPS+melatonin+nigericin”, 95% CI [3.78 to 6.71], *P* = 0.0015; group “morphine+nigericin” versus group “morphine+melatonin+nigericin”, 95% CI [4.19 to 8.12], *P* = 0.0005). (*F-G*) The BV2 cells were quantified for diameter of cell nucleus (n = 3-4 slices per group, F_4,11_ = 15.41, *P* = 0.0002; control group versus group “LPS+nigericin”, 95% CI [0.86 to 3.08], *P* = 0.0012; group “LPS+nigericin” versus group “LPS+melatonin+nigericin”, 95% CI [-2.22 to -0.0004], *P* = 0.0489; group “morphine+nigericin” versus group “morphine+melatonin+nigericin”, 95% CI [-1.95 to -0.09], *P* = 0.03). Cell nucleus was stained by DAPI (blue). The treatments in (*A*) and (*F*) were same as Fig. S3. Data shown were representative of three independent experiments with similar results. Values were presented as mean ± SD in (*E* and *G*). Group differences were analyzed by one-way ANOVA. *, *P*<0.05; **, *P*<0.01; ***, *P*<0.001.

**Figure S5.** **Lack of cross-tolerance between morphine and melatonin.** Mice (n=5 animals per group) were injected twice daily for 2 days with saline, vehicle, melatonin or morphine, respectively. (A) The antinociceptive potency of morphine did not differ between mice pretreated with melatonin or vehicle. (B) The antinociceptive potency of melatonin did not differ between mice pretreated with morphine or saline. The hot plate tests (52.5 °C) and tail flick tests (IR50) were performed at 30 min and 60 min after each injection, respectively.
